# Multilayered nucleotide organization reveals purifying selection and host-driven adaptation in CPV and FPV

**DOI:** 10.1186/s12917-026-05651-0

**Published:** 2026-06-23

**Authors:** Xili Feng, Liwen Xu, Chengqi Zhang, Jianhua Zhou, Xue Bai

**Affiliations:** 1 Key Laboratory of Special Animal Epidemic Disease, Ministry of Agriculture, Institute of Special Animal and Plant Sciences, Chinese Academy of Agricultural Sciences, Changchun, 130112 China; 2 School of Bioengineering, Northwest Minzu University, Lanzhou, 730030 China

**Keywords:** Canine parvovirus, Feline panleukopenia virus, Nucleotide organization, Evolutionary dynamic, Purifying selection

## Abstract

Since feline panleukopenia virus (FPV) is considered the most likely ancestor of canine parvovirus (CPV), comprehensive comparisons of nucleotide organization in corresponding viral genes between CPV and FPV may provide novel insights into the evolutionary dynamics underlying the divergence of these two viruses. Here, we characterize the evolutionary patterns of CPV and FPV genes across multiple levels of nucleotide organization. Both viruses exhibited highly conserved nucleotide usage at nonsynonymous sites, with Ka/Ks patterns consistent with strong purifying selection, whereas synonymous sites showed greater variability. CpG dinucleotides were markedly underrepresented across all four viral genes, suggesting host-associated selective pressure and/or intrinsic nucleotide compositional constraints. Extensive nonrandom biases in synonymous codon usage, codon neighboring nucleotide context, and codon pair usage further revealed fine-scale genomic optimization shaped by natural selection and nucleotide compositional constraints. Structural protein genes (*VP1* and *VP2*) displayed stronger codon usage bias and higher tRNA adaptation than nonstructural genes. Moreover, CPV genes showed greater translational adaptation to feline hosts than to canine hosts. These findings highlight how closely related parvoviruses exploit flexible nucleotide organization to facilitate host adaptation while maintaining essential protein functions.

**Supplementary Information:**

The online version contains supplementary material available at https://doi.org/10.1186/s12917-026-05651-0.

## Introduction

Canine parvovirus (CPV) and feline panleukopenia virus (FPV) are rapidly evolving single-stranded DNA viruses that cause two of the most important infectious diseases in dogs and cats, respectively [1]. CPV and FPV, which share similar evolutionary patterns and genomic organization, are classified within the species *Carnivore protoparvovirus* 1, genus *Protoparvovrius*, subfamily *Parvovirinae*, family *Parvoviridae* [2]. Both viruses possess a linear single-stranded DNA genome containing two open reading frames (ORFs) that encode nonstructural proteins NS1 and NS2 and the capsid proteins VP1 and VP2.The nonstructural proteins, which are involved in DNA replication, capsid assembly, and intracellular viral transport, share an identical N-terminal region; however, the C-terminal region of *NS2* arises from differential splicing and is encoded in a different reading frame [3]. Among the viral capsid proteins, VP1 and VP2 are splice variants that are identical in sequence except for a unique 143-amino-acid N-terminal region in VP1, which is essential for viral entry into host cells [4]. Notably, both VP2 and portions of NS1 are widely considered to play crucial roles in expanding host range and enhancing replication efficiency in host cells [5, 6].

FPV is thought to have been endemic in feline populations since before the beginning of the 20th century. In contrast, CPV emerged in the late 1970s as a result of an interspecies transmission event from other carnivores to dogs [7]. Although FPV, characterized by random genetic drift across its genome, is considered the most likely ancestral virus of CPV, the two viruses may have followed distinct evolutionary trajectories shaped by different evolutionary mechanisms [8]. CPV, FPV, and FPV-like viruses share more than 98% nucleotide and amino acid sequence identity [9], although CPV remains distinguishable from the others in its biological properties. Shortly after its emergence, the original CPV-2 spread rapidly worldwide and caused a pandemic in the naïve canine population [10]. Specific VP2 residues, such as 297 and 426, have been reported to be associated with host range and antigenic evolution [11–13]. CPV-2a, which exhibited an unusually high substitution rate comparable to those of rapidly evolving RNA viruses and showed clear geographic and temporal evolutionary patterns, was capable of effectively infecting both canine and feline hosts. Consequently, the CPV-2a lineage rapidly replaced the original CPV-2 lineage and became the ancestor of all CPV variants currently circulating worldwide [14, 15].This replacement may reflect a fitness advantage of CPV-2a over earlier variants, enabling more efficient infection in dogs and contributing to immune escape, vaccine failure, and subsequent increases in viral prevalence. Notably, among viruses infecting humans and other mammals, the highest levels of adaptation to host codon usage are observed in proteins that are abundantly incorporated into virions [16–21]. CPV and FPV exhibit substantial differences in host selectivity, since successful infection depends on the ability of the virus to enter host cell, hijack cellular machinery, and redirect host functions toward the efficient production of progeny virions. To comprehensively resolve the spatial and temporal genetic divergence between CPV and FPV, analyses must move beyond partial genomic segments and single-layer codon parameters, with a particular attention to host adaptation as reflected in codon usage landscapes and tRNA-mediated translational adaptation. Viral adaptation and virus-host co-evolution have traditionally been investigated through analyses of synonymous and nonsynonymous substitutions in viral genes. Because CPV and FPV lack their own transcriptional and translational machinery, viral gene expression is entirely dependent on the host cellular environment. In viruses infecting animals, such transcriptional and translational biases can enhance virion production within infected cells while simultaneously reducing recognition by the host immune system [22–25]. The genetic code is degenerate, with most amino acids encoded by multiple synonymous codons. Importantly, synonymous codons are not used at equal frequencies, giving rise to synonymous codon usage bias, a pervasive feature of nearly all genomes. This bias is not evolutionarily neutral but is associated with nucleotide composition [26, 27], mRNA stability [28, 29], translational accuracy and efficiency [30, 31], and co-translational protein folding [32]. Beyond codon usage itself, additional patterns of nucleotide organization, including mononucleotide composition, dinucleotide frequencies, codon neighboring nucleotide context, and codon pair usage, also contribute to genome evolution [17, 18, 33–37]. Among these factors, the nucleotide immediately following a codon exerts a particularly strong influence on codon usage, a phenomenon known as context-dependent codon bias (CDCB), which refers to bias in the nucleotide context flanking or neighboring synonymous codons) [37]. Codon pair bias, which reflects the nonrandom association of adjacent codons, has been shown to influence gene expression and viral fitness. Importantly, deliberate codon pair deoptimization has been widely used as an efficient strategy for rapid attenuation of diverse viruses [38–42]. Previous studies have shown that codon usage bias, codon pair organization, and dinucleotide composition can affect viral fitness, translational efficiency, and host interaction. Therefore, the sequence-level patterns identified in CPV and FPV are biologically meaningful even in the absence of direct experimental validation in the present study. Accordingly, our findings are interpreted in the context of published evidence linking codon-related features to viral adaptation and attenuation. In addition, viral adaptation to host translational machinery can be quantitatively assessed using the tRNA adaptation index (tAI), a tRNA-centered metric of gene expression efficiency based on codon usage, host tRNA availability and codon-anticodon pairing efficiencies [43]. Collectively, these sequency-level features provide valuable insights into viral adaptation and virus–host co-evolution. In this study, “multilayered nucleotide organization” refers to the hierarchical arrangement of viral coding sequences across several interrelated levels, including mononucleotide composition, dinucleotide bias, synonymous codon usage, codon neighboring nucleotide context, codon pair usage, and host-related translational adaptation. In this study, we performed a systematic analysis of multiple nucleotide usage patterns, including overall nucleotide composition, dinucleotide odds ratios, synonymous codon usage bias, CDCB, codon pair bias (CPB), and tAI, to achieve a deeper understanding of the evolutionary strategies underlying CPV and FPV adaptation to their respective hosts.

## Materials and methods

### Genome datasets for CPV and FPV

To construct genome data sets that capture both temporal and geographical diversity, full length genome sequences of CPV (*n* = 96) and FPV (*n* = 52) were retrieved from the Genbank database (https://www.ncbi.nlm.nih.gov/) (Supplementary Table S1). Only complete coding sequences were retained for downstream analyses. Sequences containing internal stop codons, lacking an initiator codon, or not terminating with a valid stop codon were excluded to ensure data quality and consistency.

### Quantification of mononucleotide usage bias in CPV and FPV genes

To characterize mononucleotide usage bias, the nucleotide compositions of the four bases (T, G, C and A) were first calculated for each viral gene (*VP1*, *VP2*, *NS1* and *NS2*), both across the entire coding region and at each of the three codon positions. According to these base frequencies, information entropy (IE) was employed to quantify the rank of mononucleotide usage bias for individual genes and codon positions [44, 45]. The *IE* value was calculated as:

$$IE=-\frac14\left(\sum_if_i\times\log_2\left(f_i\right)\right)$$

where *f*_*i*_ represents the frequency of nucleotide ‘*i*’ ( *i* = A, T, C and G). The lower *IE* values indicate the greater deviation from uniform nucleotide usage, whereas the higher *IE* values represent a more homogeneous nucleotide distribution. The *IE* values for the four viral genes (*VP1*, *VP2*, *NS1* and *NS2*) were visualized using violin plots gene rated with the ggplot2 package.

To further investigate selective pressures acting on CPV and FPV genes, synonymous (K_s_) and nonsynonymous (K_a_) substitution rates were estimated using the Nei-Gojobori method [46]. A Ka > Ks ratio greater than one indicates positive selection, a ratio close to one suggests neutral evolution; and a ratio less than one reflects purifying selection acting to conserving protein function.

### Quantification of dinucleotide usage bias in CPV and FPV genes

Following mononucleotide analysis, dinucleotide usage bias was assessed to characterize higher-order nucleotide organization in CPV and FPV genomes. Dinucleotide relative abundance (DOR), also referred to as the dinucleotide odds ratio, was calculated for all 16 possible dinucleotide combinations within each viral gene according to the formula: (DOR) = *f(xy) / f(x)f(y)*, where *f(xy)* denotes the observed frequency of dinucleotide (*xy*), and *f(x)* and *f(y)* represent the frequencies of nucleotides *x* and *y*, respectively [47]. Dinucleotide with DOR values < 0.78 or > 1.23 were considered significantly underrepresented or overrepresented, respectively; whereas values between 0.78 and 1.23 were interpreted as indicative of weak or negligible bias. To ensure the direct comparison of dinucleotide usage bias between CPV and FPV, *DOR* values for *VP1*, *VP2*, *NS1*, and *NS2* were visualized using violin plots gene rated with the ggplot2 package.

### Assessment of codon usage bias in CPV and FPV genes

Following the characterization of mononucleotide and dinucleotide usage biases, the overall synonymous codon usage bias was further assessed for CPV and FPV genes. To disentangle the relative contributions of nucleotide compositional constraint and natural selective force to synonymous codon usage patterns, an ENC vs. GC3 plot was constructed using the effective number of codons (ENC) and the GC content at the third codon position (GC3) [48]. Linear regression analysis was subsequently performed to evaluate the extent to which GC3-driven compositional constraints shape codon usage bias in individual viral genes.

According to the overall codon usage bias reflected by ENC values, the relative synonymous codon usage (RSCU) values were calculated for each viral gene. RSCU is defined as the ratio of the observed frequency of a codon to its expected frequency under equal usage of synonymous codons encoding the same amino acid [49]. Codons with RSCU values > 1.0 were considered preferentially used, whereas those with RSCU values < 1.0 were considered underused. To further classify codon usage bias intensity, codons with RSCU > 1.6 were defined as overrepresented, while codons with RSCU < 0.6 were defined as underrepresented [50]. To explore genetic diversity and clustering patterns associated with synonymous codon usages, the principal component analysis (PCA) was performed on RSCU values derived from the four viral genes of CPV and FPV. PCA, a multivariate statistical approach, reduces data dimensionality by transforming correlated variables into a set of orthogonal principal components based on covariance structure [51]. Scatter plots based on the first two principal components (*f’*_*1*_ and *f’*_*2*_ data) were constructed via GraphPad Prism 6.

### Analysis of codons neighboring nucleotide context bias in CPV and FPV genes

Following the assessment of synonymous codon usage bias, codon neighboring nucleotide context bias was further analyzed to characterize context-dependent codon usage in CPV and FPV genes. The CDCB value was calculated to quantify the relative abundance of synonymous codons in specific nucleotide contexts. For each synonymous codon *‘x*_*i*_’ encoding amino acid ‘*X*’, the CDCB value was calculated for the context *X (x*_*i*_*~n)* where ‘*X*’ denotes the amino acid, *‘x*_*i*_’ represents the *i*-th synonymous codon, and ‘*n*’ indicates the nucleotide context [52]. Previous studies have demonstrated that the nucleotide immediately following a codon (termed as N1 context) plays a dominant role in shaping CDCB [37]. Accordingly, the N_1_ context was defined as the nucleotide directly downstream of the codon and denoted as XYZ_N, where X, Y, Z, and N represent the three nucleotides of the codon and N represents the adjacent downstream nucleotide. CDCB values for the four viral genes (*VP1*, *VP2*, *NS1*, and *NS2*) of CPV and FPV were calculated to represent the relative abundance of each codon in a given N1 context. CDCB was computed according to the formula: *CDCB* = F_(*XYZ_N*)_/F_(*XYZ*)_F_(*N*)_, where F_(*XYZ_N*)_ denotes the frequency of codon (*XYZ*) followed by nucleotide N, F_(*XYZ*)_ represents the frequency of the codon, and F_(*N*)_ represents the frequency of nucleotide N in the N1 context. The higher *CDCB* values indicate the stronger bias toward a specific codon neighboring nucleotide context. Calculations for CDCB were performed following previously described procedures using Perl scripts, with reference datasets available at www.mcb.harvard.edu/gilbert/cdcb [37]. To visualize evolutionary patterns associated with codon neighboring nucleotide context, heat maps were generated based on the natural logarithm of the mean CDCB values for each codon-nucleotide combination across viral sequences.

### Analysis ofcodon pair bias in CPV and FPV genes

Following the analysis of codons neighboring nucleotide context bias, codon pair usage patterns in CPV and FPV genes were further characterized by assessing codon pair bias (CPB) (CPB describes the nonrandom preference for or avoidance of specific adjacent codon pairs). CPB quantifies the relative abundance of adjacent codon pairs by comparing the observed frequency of a specific codon pair with its expected frequency under random association. CPB was calculated according to the following formula: CPB = N_(*xyz_uvw*)_×n^2^ / [N_(*xyz*)_×N_(*uvw*)_×(n-1)]. N_(*xyz_uvw*)_ means the observed number of occurrences of a specific codon pair *xyz_uvw*, with (*xyz*) and (*uvw*) representing the upstream and downstream codons, respectively. N_(*xyz*)_ and N_(*uvw*)_ indicate the total numbers of the individual codons, and ‘*n*’ represents the total number of codons in the target gene. CPB values were calculated for all 3721 possible codon pairs (61 × 61 sense codons) across viral gene sequences. The higher CPB values indicated the stronger bias toward specific codon pair contexts. Notably, CPB is independent of both overall codon usage bias and amino acid composition. To visualize codon pair usage patterns and associated evolutionary trends, heat maps were generated based on the natural logarithm of the mean CPB values for each codon pair in the corresponding viral genes.

### Estimation of tRNA adaptation of CPV and FPV genes to hosts translation systems

To further assess the translational adaptation and expression efficiency of CPV and FPV genes in susceptible hosts, the tAI was calculated for canine and feline hosts. tAI is a quantitative measure of translational efficiency that reflects the extent to which viral codon usage is adapted to the intracellular tRNA pool of the host [43, 53, 54]. Detailed procedures for tAI calculation and host-specific tRNA datasets are provided in the Supplementary Methods. The tAI values range from 0 to 1 with lower values indicating poorer translational adaptation and reduced expression efficiency in a given host, and the higher values indicating the stronger adaptation to host tRNA availability and enhanced translational efficiency.

### Statistical analysis

Statistical analyses of nucleotide composition and information entropy data were performed using One-way ANOVA to assess differences among groups. When significant effects were detected, post hoc pairwise comparisons were conducted using the least significant difference (LSD) test. All statistical analyses were carried out using SPSS version 23.0 for Windows, and statistical significance was defined as *p* < 0.05.

## Results

### Strong purifying selection acting on nucleotide usage bias in CPV and FPV genes

**Fig. 1:**
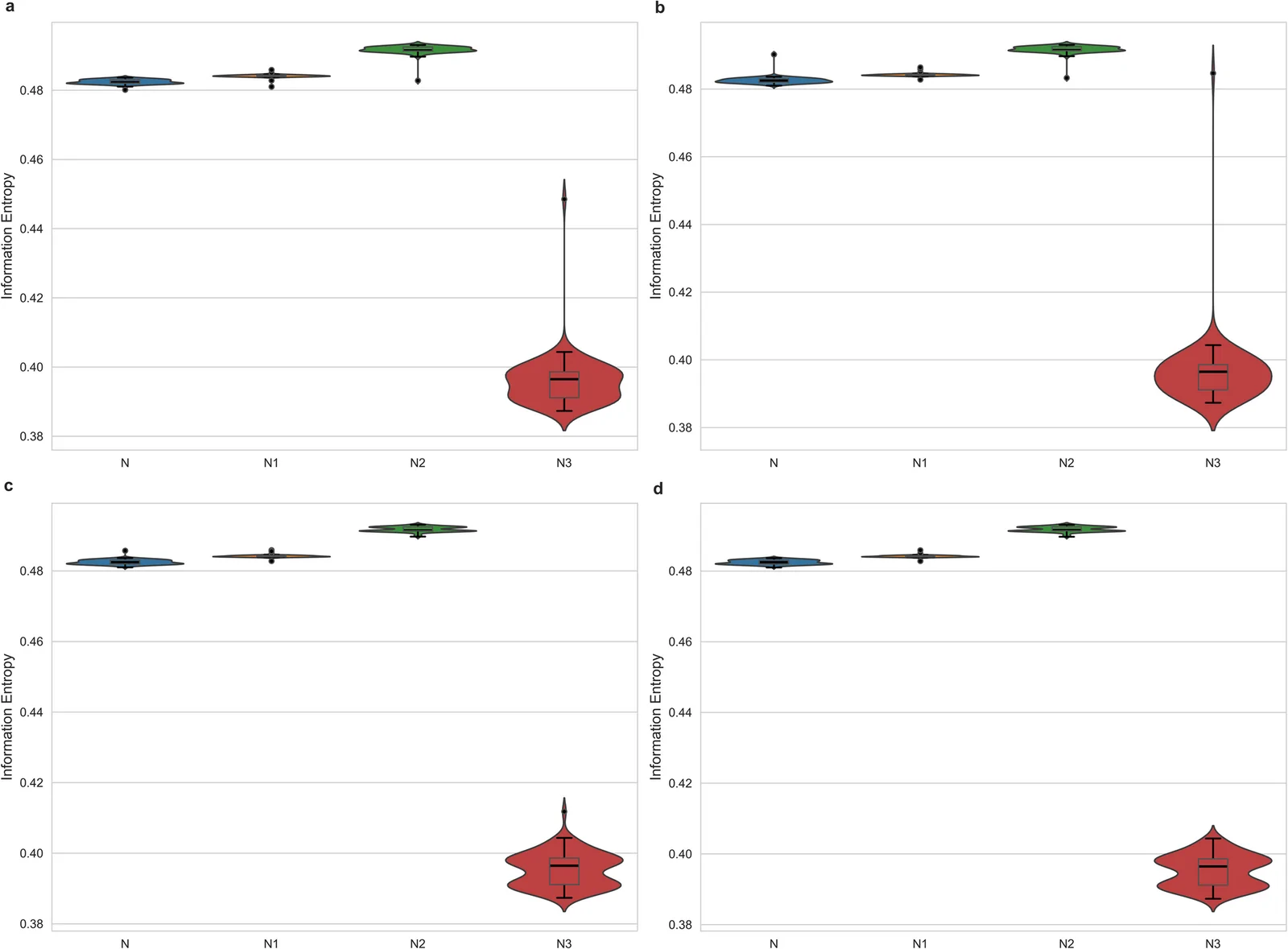
Evolutionary paradigm associated with mononucleotide usage arrangement for each viral gene of CPV illustrated by violin plot. The ‘N’ represents the whole gene, the ‘N1’, ‘N2’ and ‘N3’ mean the first, second and third codon positions, respectively. **a ***NS1* gene, **b ***NS2* gene, **c ***VP1* gene, **d ***VP2* gene

**Fig. 2:**
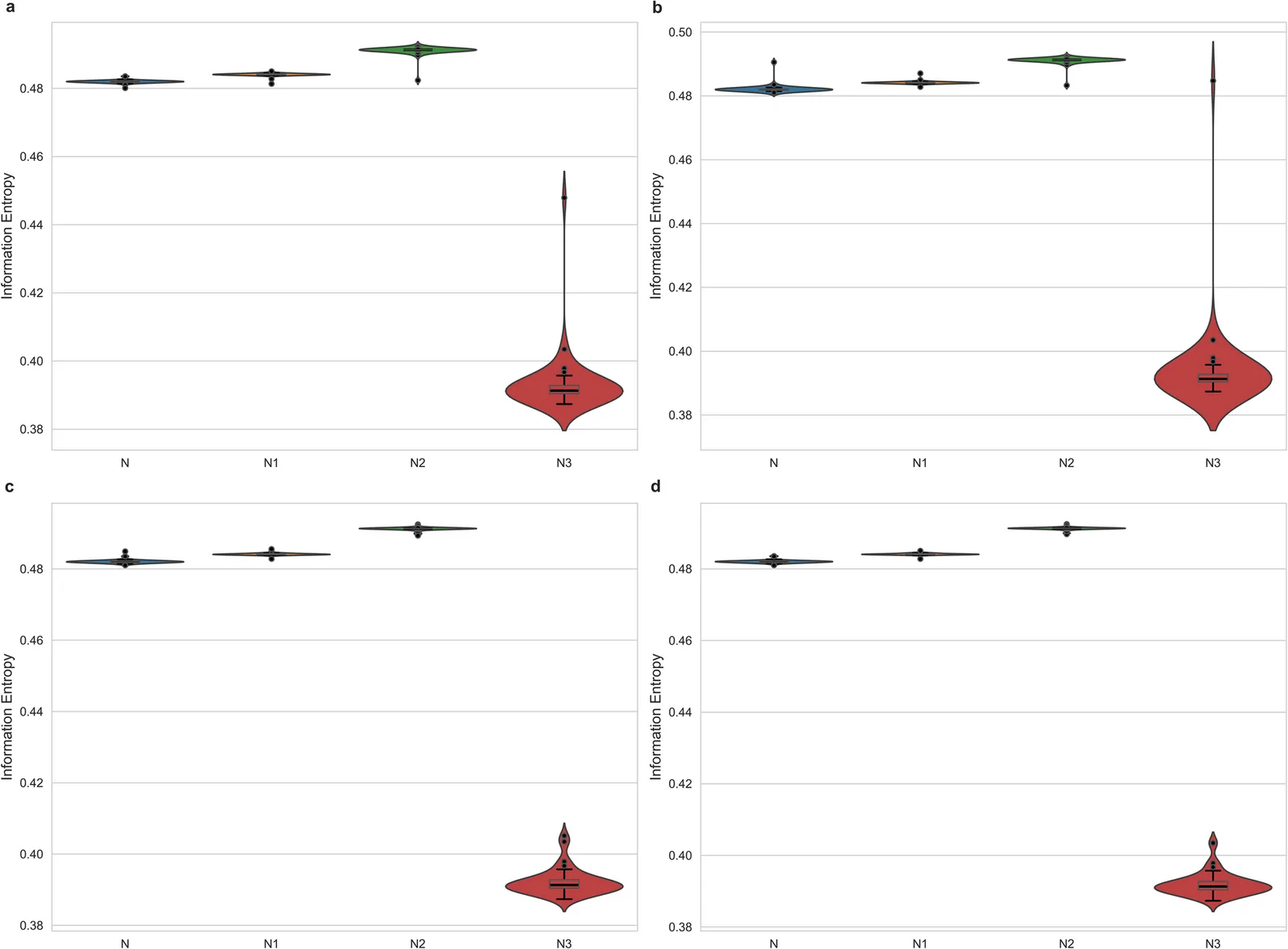
Evolutionary paradigm associated with mononucleotide usage arrangement for each viral gene of FPV illustrated by violin plot. The ‘N’ represents the whole gene, the ‘N1’, ‘N2’ and ‘N3’ mean the first, second and third codon positions, respectively. **a ***NS1* gene, **b ***NS2* gene, **c ***VP1* gene, **d ***VP2* gene

IE was used to quantify mononucleotide usage bias across the complete coding regions and individual codon positions of CPV and FPV genes. For all four CPV genes (NS1, NS2, VP1, and VP2), IE values at the first and second codon positions were consistently high and exhibited very limited variation, whereas substantially lower IE values with greater dispersion were observed at the third codon position (Fig. 1a–d). Specifically, for the CPV NS1 gene, IE values were 0.482 ± 0.001 for the whole gene, 0.484 ± 0.001 for the first codon position, 0.492 ± 0.002 for the second codon position, and 0.396 ± 0.009 for the third codon position (Fig. 1a). Similar patterns were observed for NS2 (0.483 ± 0.001 overall; 0.484 ± 0.0004 at the first position; 0.492 ± 0.001 at the second position; 0.397 ± 0.013 at the third position; Fig. 1b), VP1 (0.483 ± 0.001 overall; 0.484 ± 0.0003 at the first position; 0.492 ± 0.001 at the second position; 0.396 ± 0.005 at the third position; Fig. 1c), and VP2 (0.483 ± 0.001 overall; 0.484 ± 0.0003 at the first position; 0.492 ± 0.001 at the second position; 0.395 ± 0.005 at the third position; Fig. 1d). The markedly lower IE values at the third codon position indicate the greater variability at synonymous sites, whereas the limited variation observed at the first and second codon consistent with stronger evolutionary constraints on amino acid altering substitutions. Of note, for the *NS1*, *VP1*, and *VP2* genes of CPV, mononucleotide usage variability at the third codon position displayed a clear bimodal distribution, suggesting the presence of two genetically distinct groups associated with synonymous-site nucleotide usage (Fig. 1a, c, d). This pattern was not observed the *NS2* gene.A comparable overall pattern was observed for FPV genes. For FPV *NS1*, IE values were 0.482 ± 0.001 for the whole gene, 0.484 ± 0.001 at the first codon position, 0.491 ± 0.002 at the second codon position, and 0.394 ± 0.011 at the third codon position (Fig. 2a). Similar distributions were detected for NS2 (0.482 ± 0.002 overall; 0.484 ± 0.001 at the first codon position; 0.491 ± 0.002 at the second codon position; 0.395 ± 0.018 at the third codon position; Fig. 2b), *VP1* (0.482 ± 0.001 overall; 0.484 ± 0.0003 at the first codon position; 0.491 ± 0.001 at the second codon position; 0.392 ± 0.004 at the third codon position; Fig. 2c), and *VP2* (0.482 ± 0.0004 overall; 0.484 ± 0.0003 at the first codon position; 0.491 ± 0.0004 at the second codon position; 0.392 ± 0.003 at the third codon position; Fig. 2d). In our study, evidence for purifying selection was inferred mainly from the conservation of nonsynonymous sites and Ka/Ks patterns, whereas IE was used to describe the distribution of nucleotide usage bias across codon positions. The codon-position-specific IE patterns suggest that most variability is tolerated at sites with greater synonymous flexibility, consistent with purifying selection acting more strongly on nonsynonymous positions. Comparative analysis revealed highly similar codon-position-specific mononucleotide usage patterns between CPV and FPV across all four viral genes (Figs. 1 and 2). However, unlike CPV, the *NS1*, *VP1* and *VP2* genes of FPV did not exhibit clear genetic clustering based on synonymous-site mononucleotide usage variability, indicating subtle differences in synonymous site evolutionary dynamics between the two viruses.

Furthermore, clear divergence between the evolutionary dynamics of CPV and FPV was evident from the analysis of Ka and Ks substitution rates (Table 1). Across all four viral genes, Ks values were consistently higher than Ka values in both CPV and FPV, indicating an excess of synonymous substitutions relative to amino acid altering changes. Correspondingly, Ka/Ks ratios for each gene were uniformly below one, suggesting that purifying selection predominates over positive selection in shaping the evolution of these viral genes. These Ka and Ks patterns are highly consistent with the mononucleotide usage bias observed at different codon positions. Specifically, the strong constraint at nonsynonymous sites inferred from low Ka values parallels the limited variability in mononucleotide usage at the first and second codon positions, whereas the elevated Ks values are consistent with the greater variability observed at synonymous sites. Together, these findings indicate that purifying selection is a major force shaping protein-coding evolution in both CPV and FPV.

**Table 1:** Ratio of nonsynonymous and synonymous substitutions for the given viral gene

	CPV			FPV		
	^a^Ka value	^b^Ks value	Ka/Ks	^a^Ka value	^b^Ks value	Ka/Ks
NS1	0.003	0.027	0.111	0.004	0.025	0.160
NS2	0.007	0.023	0.304	0.008	0.010	0.800
VP1	0.005	0.017	0.294	0.003	0.026	0.115
VP2	0.005	0.017	0.294	0.003	0.026	0.115

^a^ the mean value calculated by all comparison data derived from the corresponding viral gene

^b^ the mean value calculated by all comparison data derived from the corresponding viral gene

### Strong suppression of CpG dinucleotide usage in CPV and FPV genes

**Fig. 3:**
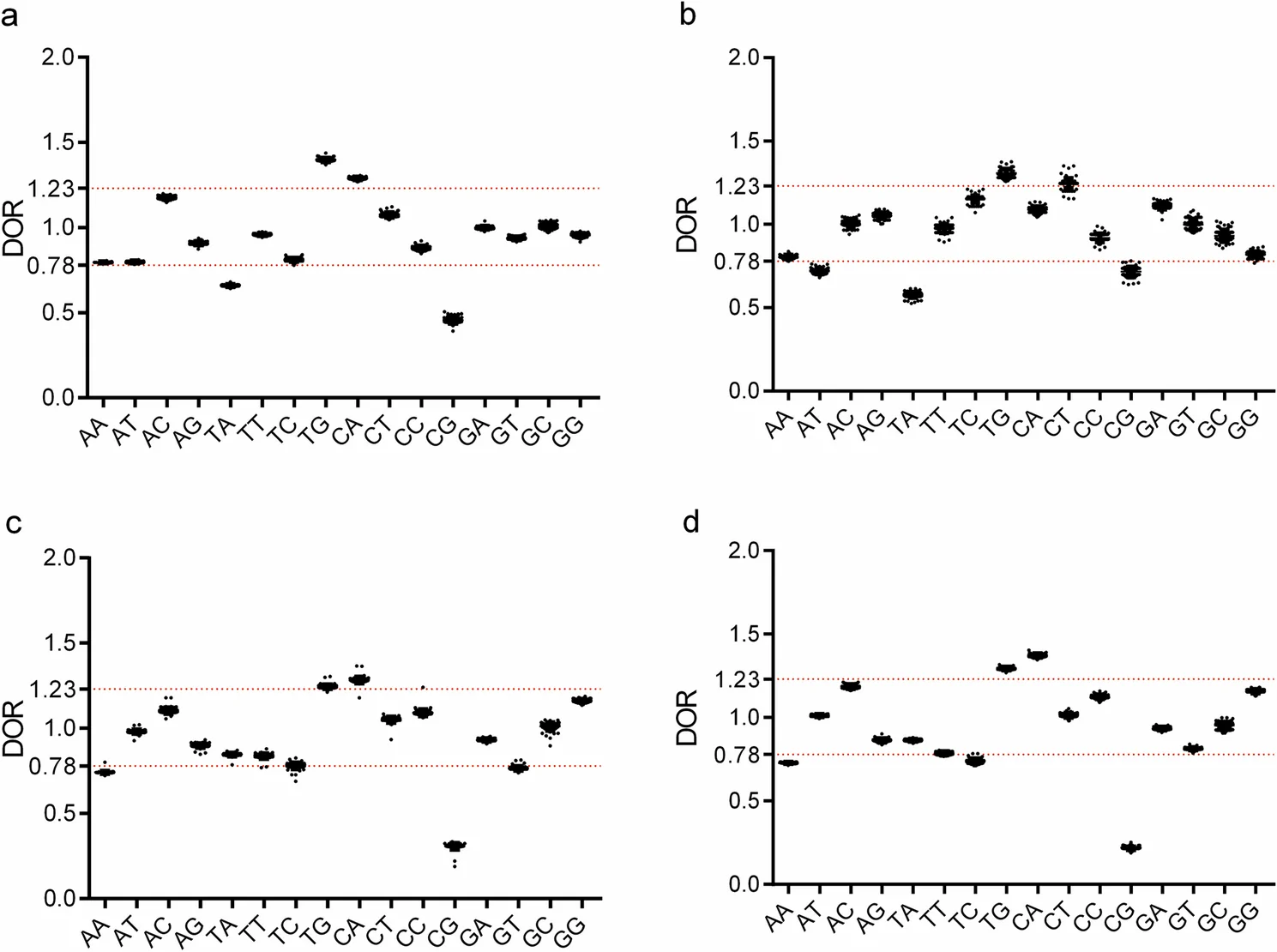
Variations of dinucleotide odds ratio for viral genes of CPV illustrated by scatter plot. The two red broken lines mean the value of 1.23 and 0.78. **a ***NS1* gene, **b ***NS2* gene, **c ***VP1* gene, **d ***VP2* gene

**Fig. 4:**
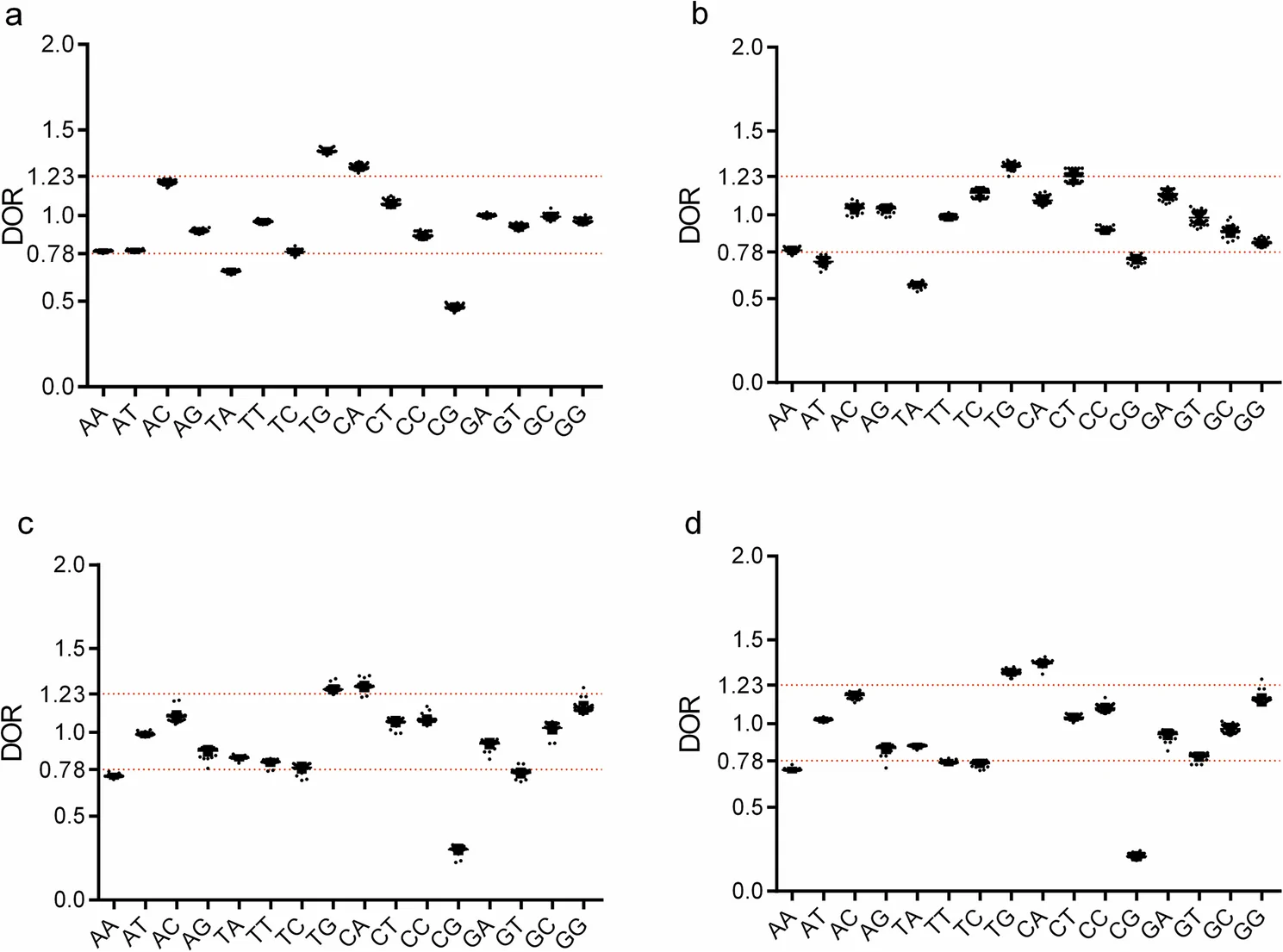
Variations of dinucleotide odds ratio for viral genes of FPV illustrated by scatter plot. The two red broken lines mean the value of 1.23 and 0.78. **a ***NS1* gene, **b ***NS2* gene, **c ***VP1* gene, **d ***VP2* gene

Following the characterization of mononucleotide usage patterns (Figs. 1 and 2), dinucleotide usage bias was further examined by analyzing DORs for all 16 possible dinucleotide combinations in CPV and FPV genes. Considerable variability in DOR values was observed among genes, whereas CPV and FPV exhibited highly similar dinucleotide usage profiles within corresponding gene (Figs. 3 and 4). Among all dinucleotides, CpG was consistently the most underrepresented dinucleotide (DOR < 0.78) in all four viral genes of both CPV and FPV, indicating pronounced CpG suppression across the genomes of the two viruses. In addition, the dinucleotide TA was underrepresented in the *NS1* and *NS2* genes but not in the *VP1* and *VP2* genes of either virus (Figs. 3 and 4). In contrast, several dinucleotides were overrepresented (DOR > 1.23) in a gene-specific manner. Specifically, TG and CA were overrepresented in the *NS1* and *VP2* genes, TG was overrepresented in the *NS2* gene, and CA was overrepresented in the *VP1* gene of both CPV and FPV. Despite these shared patterns, a notable difference was observed in the *VP1* gene, where the dinucleotide TG was overrepresented in CPV but underrepresented in FPV, indicating subtle divergence in dinucleotide usage bias between the two viruses at the gene-specific level.

### Similar synonymous codon usage patterns in CPV and FPV genes

Overall, CPV and FPV exhibited highly similar synonymous codon usage patterns across all four viral genes; In both viruses, preferred synonymous codons (RSCU > 1.0) were predominantly A/T-ended, whereas non-preferred codons (RSCU < 1.0) were more frequently C or G ended, suggesting a strong influence of nucleotide composition on synonymous codon usage (Tables 2 and 3). Consistent patterns of overrepresented synonymous codons were observed between CPV and FPV in a gene-specific manner. In the *NS1* gene, overrepresented codons included ACT and ACA (Thr), GGT and GGA (Gly), GAA (Glu), CAA (Gln), TGT (Cys), ATT (Ile), GTT (Val), TTT (Phe), TTA (Leu), CCA (Pro), AGT (Ser), GCA (Ala), and AGA (Arg). In the *NS2* gene, overrepresented codons comprised CCT and CCA (Pro), CAA (Gln), GTT (Val), ATT (Ile), CAT (His), GGA (Gly), GCA (Ala), and AGA (Arg). For the *VP1* and *VP2* genes, shared overrepresented codons included GAA (Glu), GTT and GTA (Val), GGT (Gly), GAT (Asp), TAT (Tyr), CAT (His), TCT (Ser), AAA (Lys), ACT and ACA (Thr), TTT (Phe), CAA (Gln), TGT (Cys), ATT (Ile), GCA (Ala), TTA (Leu), CCA (Pro), and AGA (Arg), with AGT (Ser) additionally overrepresented in VP1 (Tables 2 and 3). Conversely, a largely overlapping set of underrepresented synonymous codons was identified in both viruses. These included multiple C- or G-ended codons across amino acids, such as AAG (Lys), ACC and ACG (Thr), AGC, TCC, and TCG (Ser), ATC (Ile), CAC (His), CAG (Gln), CCC and CCG (Pro), CGA and CGG (Arg), CTC and CTG (Leu), GAG (Glu), GCC, GCG, and GCT (Ala), GGC and GGG (Gly), GTC (Val), TAC (Tyr), TGC (Cys), and TTC (Phe) in the *NS1* gene. Similar underrepresentation patterns were observed in the *NS2* and *VP1 / VP2* genes, with minor gene-specific differences detailed in Tables 2 and 3. Notably, several synonymous codons were entirely absent or rarely used in specific genes of both viruses. For example, CGT and AGG (Arg) were not selected in the NS1 gene; CTG (Leu), ATA (Ile), CCC and CCG (Pro), GCT (Ala), CGG and AGG (Arg), and GGT (Gly) were not selected in the *NS2* gene; and CTC (Leu), CGA and AGG (Arg) were not selected in the *VP2* gene. In addition, synonymous codons containing the CpG dinucleotide, which is known to stimulate host innate immune responses, were consistently suppressed in both viruses, whereas synonymous codons containing the TA dinucleotide did not display an equivalent level of suppression (Tables 2 and 3). These observations further support the presence of strong and stable selective constraints shaping synonymous codon usage in CPV and FPV.

**Table 2:** No selection of codon neighboring a particular nucleotide context in the given viral gene of CPV and FPV

Viral gene	Codon neighboring a particular nucleotide context
NS1 gene of CPV	CCC_T; CCC_C; CCC_G; GCC_C; GCC_G
NS2 gene of CPV	ATA_A; ACC_C; TAT_C; TAG_G; TCC_C; TCG_A; TGC_T; TGC_C; CAG_G; CTA_G; CCC_G; CGC_G; CGG_T; GTC_G; GCT_T; GCC_C; GCC_G; GCG_C; GGT_A; GGT_G; GGC_C; GGG_T; GGG_G
VP1 gene of CPV	TCG_G; CGC_G
VP2 gene of CPV	ACC_G; ACG_C; TTC_G; TCC_G; TCG_C; TCG_G; CAC_G; CTC_G; CCG_A; CCG_T; CGC_G; CGG_C; GCT_C; GCC_G
NS1 gene of FPV	ACC_C; TGC_C; CCC_C; CCC_G; CCG_C; GTC_G
NS2 gene of FPV	ATA_A; ATA_T; ACC_C; ACG_A; TAT_C; TAG_C; TAG_G; TCC_C; TCG_A; TGC_T; TGC_C; TGC_G; CAT_A; CAG_G; CTA_C; CTA_G; CCC_A; CCC_T; CCC_C; CCC_G; CCG_C; CGC_G; CGG_T; GAT_T; GTC_G; GTG_T; GCT_T; GCT_C; GCC_C; GCC_G; GCG_C; GGT_A; GGT_G; GGC_T; GGC_C; GGG_T; GGG_G
VP1 gene of FPV	TCG_A; GCG_A
VP2 gene of FPV	TCG_A; CAC_G; CTC_G; CCG_T; CGA_T; CGA_C; CGT_A; GCT_C; GCC_C; GCC_G; GCG_A

**Table 3:** Comparison of tRNA adaptation of viral genes to different hosts between CPV and FPV

	CPV	FPV	Significance
NS1 gene in canine	0.181 ± 0.002	0.181 ± 0.002	*p* value > 0.05
NS2 gene in canine	0.186 ± 0.003	0.186 ± 0.004	*p* value > 0.05
VP1 gene in canine	0.205 ± 0.002	0.202 ± 0.004	*p* value < 0.001
VP2 gene in canine	0.216 ± 0.002	0.213 ± 0.003	*p* value < 0.001
NS1 gene in feline	0.214 ± 0.002	0.214 ± 0.002	*p* value > 0.05
NS2 gene in feline	0.210 ± 0.003	0.208 ± 0.003	*p* value < 0.05
VP1 gene in feline	0.225 ± 0.002	0.222 ± 0.004	*p* value < 0.001
VP2 gene in feline	0.238 ± 0.002	0.234 ± 0.003	*p* value < 0.001

**Fig. 5:**
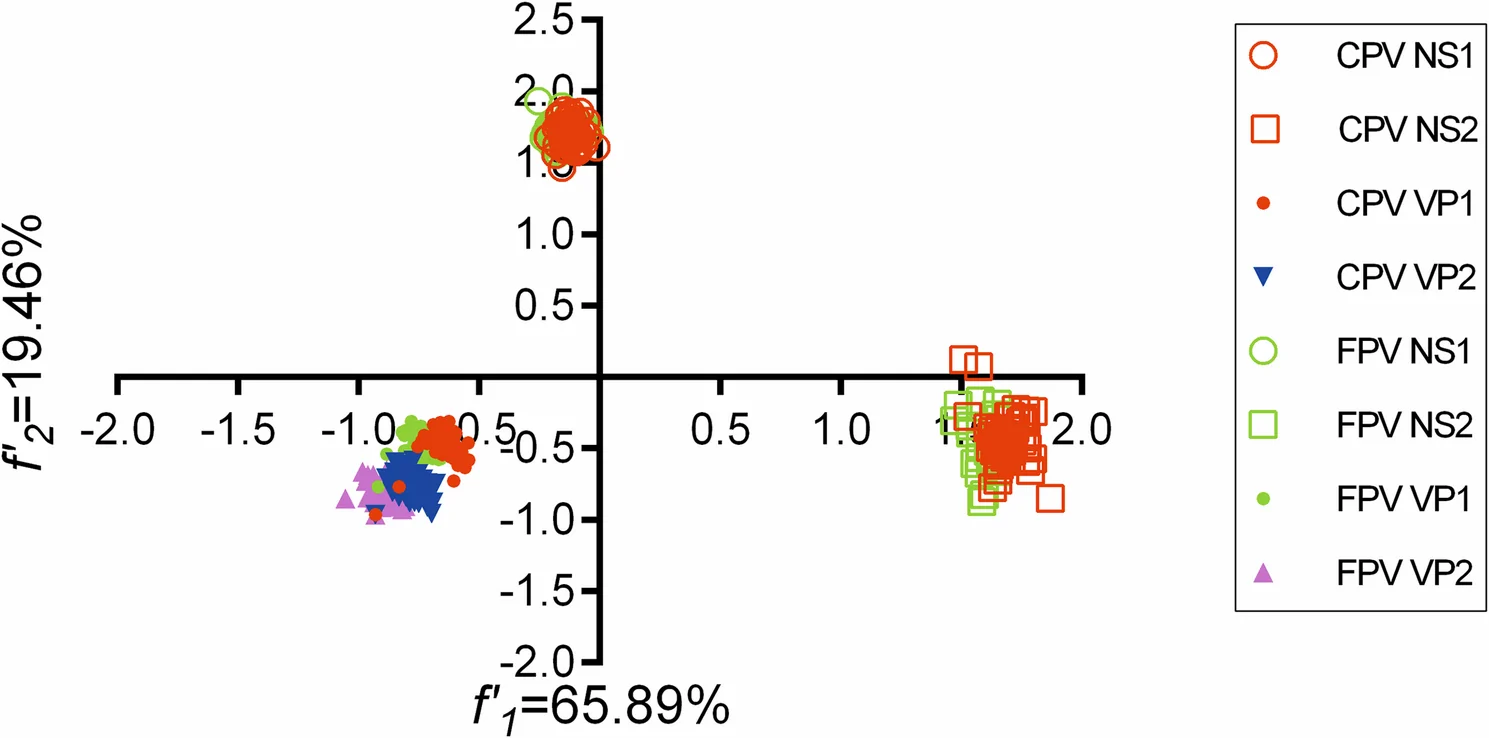
Evolutionary paradigm associated with synonymous codon usage variations for viral genes of CPV and FPV illustrated by PCA

To further explore the relationship between synonymous codon usage and viral protein function, PCA was performed based on RSCU values for the four viral genes (Tables 2 and 3), PCA revealed that *VP1* and *VP2*, which encode structural proteins, clustered closely and shared highly similar synonymous codon usage patterns, whereas *NS1* and *NS2*, encoding nonstructural proteins, exhibited more divergent patterns (Fig. 5).This separation suggests that evolutional factors associated with protein function, in addition to nucleotide composition, contribute to synonymous codon usage patterns in CPV and FPV.

### Codon usage bias in CPV and FPV genes is shaped by both natural selection and compositional constraints

**Fig. 6:**
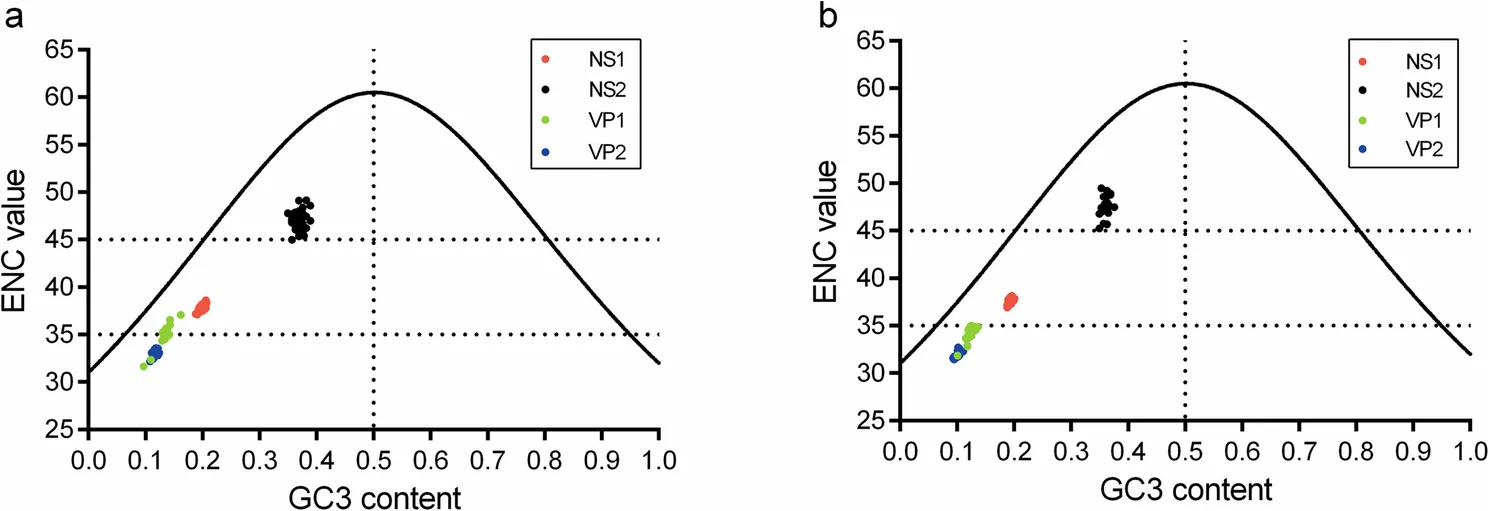
The plot composed of ENC vs. GC3 content for each viral gene of both CPV and PFV. **a **the four viral genes of CPV; **b **the four viral genes of FPV

**Fig. 7:**
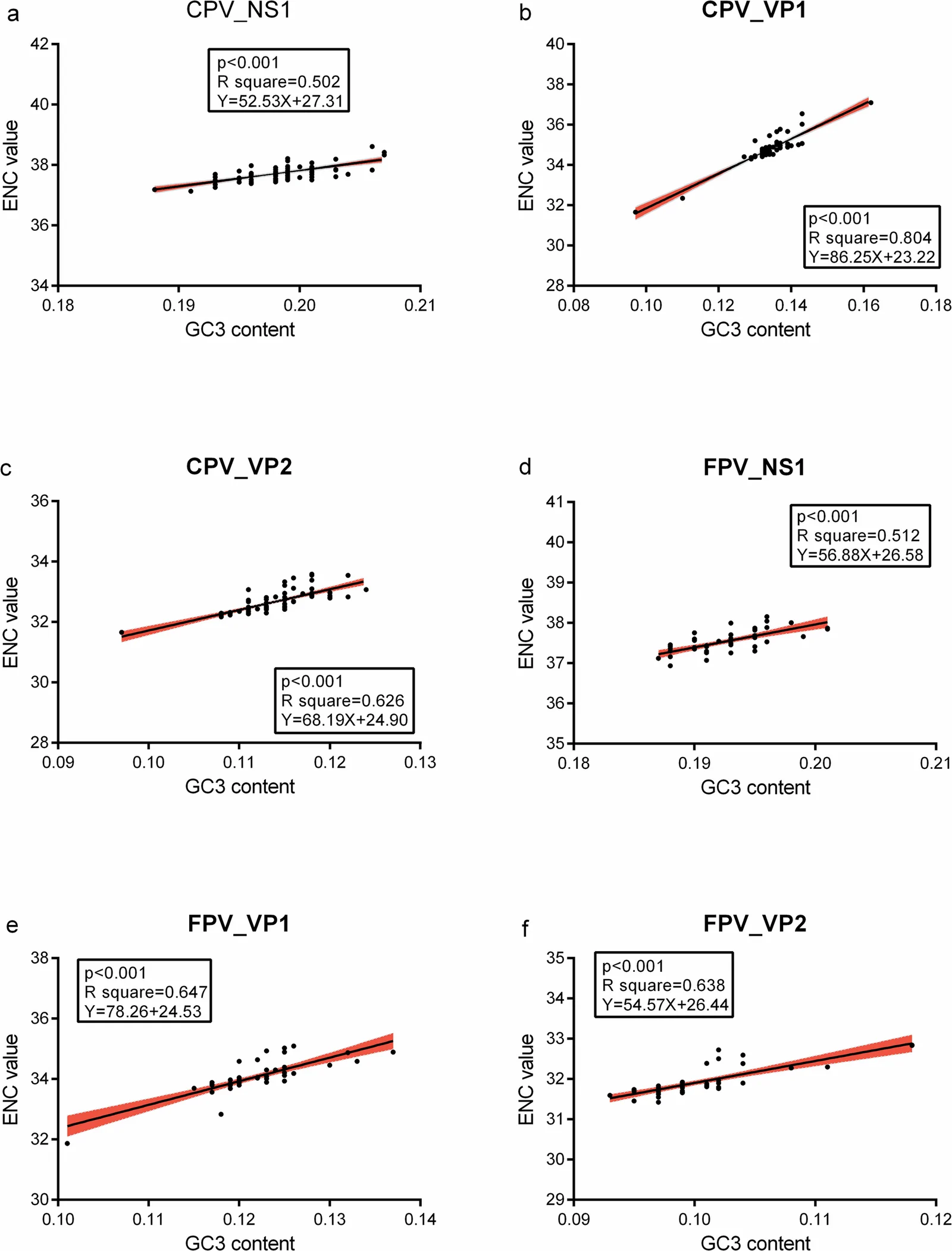
The linear relationships between ENC value and GC3 content in viral genes for *NS1*, *VP1* and *VP2* in both CPV and FPV. The red regions reflect 95% confidential interval. **a ***NS1* in CPV; **b ***VP1* in CPV; **c ***VP2* in CPV; **d ***NS1* in FPV; **e ***VP1* in FPV; **f ***VP2* in FPV

To evaluate the relative contributions of nucleotide composition constraint and natural selection to overall codon usage bias, ENC vs. GC3 plots were constructed for the four viral genes of CPV and FPV. Genes located below the expected ENC curve suggest that mutation pressure alone cannot fully explain codon usage patterns and that additional selective and/or compositional constraints are involved. Because ENC deviation cannot distinguish translational selection from other constraints by itself, we interpreted these results together with dinucleotide patterns, codon context bias, codon pair bias, and tAI-based host adaptation estimates. The stronger codon usage bias and higher tAI values observed in *VP1* and *VP2* further support the possibility that translational adaptation contributes to the codon usage pattern of structural genes (Fig. 6). Despite this shared trend, clear differences in the strength of overall codon usage bias were observed among viral genes. In both CPV and FPV, the *VP1* and *VP2* genes exhibited stronger overall codon usage bias than the *NS1* and *NS2* genes, while the *NS1* gene showed a more pronounced bias than the *NS2* gene (Fig. 6). These results suggest gene-specific differences in the balance between selective pressure and compositional constraint. Correlation analyses further revealed gene-dependent relationships between GC3 content and ENC values. Significant positive correlations were observed for the *NS1*, *VP1 and* VP2 genes, indicating that nucleotide composition constraint plays an important role in shaping overall codon usage bias in these genes (Fig. 7). In contrast, no significant correlation was detected for the *NS2* gene of CPV (*p* > 0.05) and only a very weak correlation was observed for the *NS2* gene of FPV (R^2^ < 0.1, *p* < 0.05). Accordingly, linear regression models were constructed for the *NS1*, *VP1* and *VP2* genes to further quantify the effect of GC3-driven compositional constraints. Among all viral genes examined, the CPV *VP1* gene showed the strongest association between GC3 and ENC, with an R^2^ value of 0.804, whereas the remaining genes exhibited weaker correlations (R² < 0.700) (Fig. 7), This result indicates that nucleotide compositional constraints play a particularly prominent role in shaping the overall codon usage bias of the CPV *VP1* gene.

### Strong codon neighboring nucleotide context bias in CPV and FPV genes

Based on the mononucleotide usage patterns observed at different codon positions (Figs. 1 and 2), codon neighboring nucleotide context bias was further examined in CPV and FPV genes. Context-dependent CDCB analysis revealed substantial variability across viral genes in both viruses (Figures S1–S8). Despite this variability, CPV and FPV exhibited highly similar evolutionary paradigms with respect to codon neighboring nucleotide context within each corresponding viral gene. Across all genes analyzed, codons associated with specific N1 contexts, particularly XXC_G and XXT_A (where ‘X’ represents any nucleotide), were generally weakly selected or rarely used in both CPV and FPV. In addition, several codons associated with a particular nucleotide context were entirely absent or infrequently selected in viral genes of both viruses (Table 2), indicating strong context-dependent constraints on codon usage. Consistent with these observations, overrepresented synonymous codons identified in CPV and FPV (Table S2 and S3) also exhibited pronounced bias in their neighboring nucleotide context (Figures S1-S8). Together, these findings indicate that codon neighboring nucleotide context is highly nonrandom and likely contributes to the evolution of dinucleotide and synonymous codon usage patterns in both CPV and FPV.

### Nonrandom codon pair bias in CPV and FPV genes

Building on the observed evolutionary patterns of synonymous codon usage bias and codon neighboring nucleotide context bias (Tables S2 and S3; Figures S1-S8), codon pair usage patterns were further examined in CPV and FPV genes. CPB scores were calculated for all 3721 possible codon pair combinations across the four viral genes of both viruses. Although CPB values exhibited a wide range of variability, the majority of codon pairs were not selected by viral genes, indicating strong nonrandom constraints on codon pair usage. In CPV, 77.83% (2896/3721), 92.10% (3427/3721), 77.21% (2873/3721) and 81.91% (3048/3721) of codon pairs were not selected by the *NS1*, *NS2*, *VP1* and *VP2* genes, respectively (Figures S9-S12).Similarly, in FPV, 80.46% (2994/3721), 93.95% (3496/3721), 78.53% (2922/3721) and 82.77% (3080/3721) of codon pairs were not selected by the *NS1*, *NS2*, *VP1* and *VP2* genes, respectively (Figures S13-S16). These results indicate that codon pair usage in both viruses is highly constrained and nonrandom. Among the limited set of preferred codon pairs (defined as log-transformed CPB values > 2.0), several were shared between CPV and FPV in a gene-specific manner. Specifically, ATC_GCC, AGC_GCT, GGG_CGC and GCC_CTC were preferentially used in the *NS1* gene (Figures S9 and S13), CGA_GTC and GCC_CTC in the *NS2* gene (Figures S10 and S14), TCG_TTC, ACG_CAG and AGG_GCC in the *VP1* gene (Figures S11 and S15) and ACG_CAG, AGC_TCA and GGG_CGG in the *VP2* gene (Figures S12 and S16). According to synonymous codon usage patterns of viral genes of the two viruses (Tables S2 and S3), many of these preferred codon pairs consisted predominantly of synonymous codons that were individually underrepresented in CPV and FPV genes (Tables S2 and S3). Together, these findings demonstrate that codon pair usage in CPV and FPV is strongly nonrandom and influenced by constraints operating beyond individual codon preferences, reflecting higher-order organization of viral coding sequences.

### Structural genes exhibit higher host tRNA adaptation than nonstructural genes

Based on the comprehensive analyses of nucleotide usage bias, synonymous codon usage, codon neighboring nucleotide context, and codon pair described above, tAI was further evaluated for each viral gene of CPV and FPV to assess translational adaptation to their respective hosts (canine and feline). Overall, tAI values revealed clear gene- and host-specific patterns of translational adaptation (Table 3). Across both viruses, structural protein genes (*VP1* and *VP2*) consistently exhibited higher levels of tRNA adaptation than nonstructural protein genes (*NS1* and *NS2*), regardless of host species. Moreover, viral genes generally exhibited higher tRNA adaptation to feline hosts than to canine hosts for both CPV and FPV, indicating enhanced translational compatibility with the feline cellular environment. When comparing host-specific adaptation between the two viruses, similar levels of tRNA adaptation to canine hosts were observed for the nonstructural protein genes of CPV and FPV. In contrast, the structural protein genes of CPV displayed higher tRNA adaptation to canine hosts than those of FPV; A comparable pattern was observed for adaptation to feline hosts: while the *NS1* gene showed similar tRNA adaptation levels in both viruses, the *NS2* gene and structural protein genes of CPV exhibited higher tRNA adaptation to feline hosts than their FPV counterparts (Table 3). Taken together, these results indicated that although CPV and FPV share highly similar evolutionary features of synonymous codon usage (Tables S2 and S3), structural protein genes possess superior translational adaptation to host tRNA pools compared with nonstructural genes. Moreover, CPV genes generally exhibited higher translational adaptation to feline hosts than to canine hosts, highlighting subtle but meaningful differences in host adaptation between the two viruses.

## Discussion

This study provides a comprehensive overview of the evolutionary patterns shaping nucleotide organization in the genomes of CPV and FPV. Although CPV has a relatively short evolutionary history in canine hosts compared with FPV in felines hosts, and although the CPV-2a lineage has acquired a broader host range, both viruses maintained remarkably stable nucleotide compositions across their four viral genes. This stability was particularly evident at the first and second codon positions, which are more closely associated with nonsynonymous changes (Figs. 1 and 2). These conserved patterns indicate strong purifying selection acting to preserve protein structure and function. In this study, Ka/Ks analysis was performed at the gene level to estimate overall selective constraints. However, confidence intervals and site-specific selection analyses were not included; therefore, future studies should incorporate these approaches to refine the evolutionary inference. Although CPV differs from FPV by less than 1% at the nucleotide sequence level, limited genetic changes have enabled CPV to expand its host range and became a widespread pathogen in previously resistant host species [7]. Geographic and temporal heterogeneity among CPV populations may have further contributed to viral competition, local differentiation, and the current epidemiological landscape of CPV. Consistent with this possibility, CPV exhibited distinct mononucleotide usage patterns at the third codon position in the NS1, VP1 and VP2 genes compared with FPV (Figs. 1 and 2), suggesting divergence in synonymous-site evolution between the two viruses. The bimodal distribution observed at third codon positions in some CPV genes may indicate underlying heterogeneity within the CPV dataset, potentially associated with subtype divergence, temporal structure, or geographic sampling differences. Because the present analysis was not stratified by lineage, location, or collection time, the relative contribution of these factors could not be determined. Moreover, although the genome datasets were assembled to capture temporal and geographical diversity, uneven regional representation in publicly available sequences may still introduce sampling bias.Such bias could influence the observed distribution of nucleotide usage patterns, especially for features sensitive to lineage structure or population history. Mononucleotide usage bias at synonymous sites generally considered to reflect mutational pressure, whereas nucleotide usage at the first and second codon positions is more strongly constrained by amino acid requirements and protein function. These position-specific constraints may further influence higher-order genomic features, including dinucleotide usage patterns. In this study, pronounced deviations in dinucleotide usage were observed across all viral genes of both CPV and FPV (Figs. 3 and 4), indicating that viral genomic architecture is shaped by forces beyond single-nucleotide composition. Dinucleotide frequency and arrangement can influence local DNA biomechanical properties, including flexibility, bendability, and susceptibility to deformation. In CPV and FPV genes, several AT-rich dinucleotides, including AA, AT, TA and TT, showed reduced usage (Figs. 3 and 4). Runs of AT or AA / TT dinucleotides are known to promote DNA bending and nucleosome positioning, whereas CG-rich regions tend to form more rigid structures. Periodic distributions of specific dinucleotides (e.g., AA, TT, TA) within nucleosomal DNA are associated with histone octamer binding preferences and may modulate chromatin accessibility [55, 56]. In addition, transcription factor binding sites often display characteristic nucleotide contexts at their boundaries, which may facilitate DNA bending or unwinding during transcription initiation [57]. Beyond structural effects, dinucleotide composition also affects viral evolution and host interactions. In particular, the strong suppression of CpG and TpA dinucleotides in CPV and FPV genes may reflects selective pressures imposed by host antiviral defenses. CpG suppression is widely recognized as a viral strategy associated with evasion of innate immune mechanisms that preferentially target non-self RNA enriched in CpG motifs [58, 59]. Moreover, CpG and TpA frequencies can modulate viral replication efficiency and translational output, thereby influencing viral fitness and pathogenicity [60–62]. In addition to CpG depletion, variation in TA representation among genes may reflect gene-specific compositional bias and coding constraints. Collectively, these findings indicate that the genomic organization of CPV and FPV is shaped by the interplay among mutational bias, purifying selection, and host-driven selective pressures. This multilevel constraint acts on mononucleotide composition, dinucleotide usage, and higher-order sequence organization, ultimately contributing to viral adaptation, host range evolution, and pathogenic potential. However, because this study was based on sequence-level analyses, it remains unclear whether CpG depletion in CPV and FPV is directly driven by specific host antiviral effectors, such as ZAP, or instead reflects long-term mutational and compositional biases.

Mononucleotide and synonymous codon usage biases are influenced by multiple factors, including host-specific requirements and protein functional constraints [45, 63]. Codon usage bias, defined as the preferential use of certain synonymous codons, is generally shaped by mutation pressure, natural selection, and genetic drift. The strong synonymous codon usage biases observed in CPV and FPV genes suggest that selective forces related to the host cellular environment, host–virus interactions, and intrinsic immune responses may contribute to the evolution of viral coding sequences (Tables S2 and S3).Translational selection and codon usage bias are particularly evident in unicellular organisms and viruses, where preferred synonymous codons are closely linked to translation efficiency and the fine-tuning of protein synthesis [64–66]. Comparison of synonymous codon usage between CPV and FPV showed that the NS1 gene showed the most similar evolutionary patterns in both viruses (Figs. 5 and 6). In addition, structural protein genes (VP1 and VP2) exhibited more consistent synonymous codon usage patterns than the nonstructural genes (NS1 and NS2), suggesting that protein function substantially influences codon usage. Moreover, CPV genes showed enhanced translational adaptation to feline hosts than to canine hosts. The higher feline-related tAI values observed in CPV genes are consistent with the proposed FPV-like ancestral origin of CPV and may also help explain the cross-host capability reported for some CPV lineages. However, these tAI patterns should be interpreted as comparative translational compatibility signals rather than direct functional measurements of host range or replication efficiency. In addition, because the present tAI analysis was conducted at the species level and did not incorporate tissue-specific tRNA abundance, the observed pattern should be interpreted cautiously. Synonymous codon changes can affect mRNA stability, translation speed, and nascent polypeptides folding, thereby influencing protein function [30, 67–72]. Apart from natural selection, nucleotide composition constraints play an important role in shaping synonymous codon usage patterns, particularly in the *NS1*, *VP1* and *VP2* genes of both CPV and FPV (Fig. 7). Some single-stranded DNA viruses, including CPV and FPV, evolve rapidly under these compositional constraints, with substitution rates estimated at approximately 10^− 4^ subs/site/year, similar to those observed in RNA viruses [73]. The distinct codon usage and tAI profiles of *NS2* may be related to its unique expression strategy and genomic organization. Because *NS2* is produced through alternative splicing and differs functionally from the structural proteins, its nucleotide sequence may be subject to a different balance of coding, regulatory, and translational constraints than *NS1*, *VP1*, and *VP2*.The strong bias in codon neighboring nucleotide context observed in each viral gene further reflects the combined effects of nucleotide compositional constraints and natural selection (Figures S1-S8). This finding suggests that neighboring nucleotide context may influence codon pair usage and thereby contribute to viral genome organization. Notably, most absent codon pairs and strongly biased codon pairs were composed predominantly of underrepresented synonymous codons, supporting the view that codon pair usage is shaped by constraints beyond individual codon preference (Figures S9-S16). Changes in codon pair contexts, influenced by CDCB, may affect viral protein expression and, consequently, viral fitness [35, 74]. Codon pair contexts has also been shown to contribute to phylogenetic classification across domains of life, including Eukaryota, Archaea, and Bacteria [75, 76]. However, because CPV and FPV possess compact genomes, the underrepresentation of some codon pairs may partly reflect genome-size-related compositional and coding constraints. Therefore, codon pair bias in these viruses is more appropriately interpreted as the combined outcome of genome architecture and selective pressures rather than direct evidence of active selection alone. Overall, the nucleotide organization of CPV and FPV reflects multilevel evolutionary constraints acting on mononucleotide composition, dinucleotide usage, synonymous codon usage, codon neighboring nucleotide context, and codon pair preference. These constraints differ among viral genes and appear to be associated with protein function, expression strategy, host adaptation, and genome architecture. The distinct nucleotide arrangement patterns observed across the four viral genes suggest multiple evolutionary trajectories in CPV and FPV, ultimately contributing to viral replication, adaptation, and host range evolution.

## Conclusion

This study provides a systematic analysis of nucleotide organization and codon-related evolutionary patterns in CPV and FPV. Despite their close genetic relationship, both viruses exhibit multilayered evolutionary constraints shaped by strong purifying selection, nucleotide compositional bias, and host-driven selective pressures. Conserved nucleotide usage at nonsynonymous sites reflects functional constraints on viral proteins, whereas greater variability at synonymous sites underlies lineage-specific divergence. Extensive suppression of CpG dinucleotides, together with nonrandom biases in synonymous codon usage, codon neighboring nucleotide context, and codon pair usage, indicates fine-scale genomic optimization likely driven by host immune pressure and translational selection. Structural protein genes consistently display stronger codon usage bias and higher tRNA adaptation than nonstructural genes, highlighting their central role in viral fitness and host adaptation. Collectively, these findings demonstrate how closely related DNA viruses exploit flexible nucleotide arrangements to achieve efficient host adaptation while maintaining essential protein functions.

## Supplementary Information

Supplementary Material 1.

## Data Availability

The data supporting the findings of this study are available within the article and the data that support the findings of this study are available in Science Data Bank at: https://doi.org/10.57760/sciencedb.37210.

## Abbreviations

A: Adenine
C: Cytosine
CDCB: Context-dependent codon bias
CPB: Codon pair bias
CPV: Canine parvovirus
DNA: Deoxyribonucleic acid
DOR: Dinucleotide relative abundance
ENC: Effective number of codons
FPV: Feline panleukopenia virus
G: Guanine
IE: Information entropy
Ka: Nonsynonymous substitution rate
Ks: Synonymous substitution rate
LSD: Least significant difference
NS: Nonstructural protein
ORFs: Open reading frames
PCA: Principal component analysis
RNA: Ribonucleic acid
RSCU: Relative synonymous codon usage
T: Thymine
tAI: tRNA adaptation index
tRNA: Transfer ribonucleic acid
