# Supplementary material for: Multilayered nucleotide organization reveals purifying selection and host-driven adaptation in CPV and FPV
Source: BMC Vet Res. 2026 Jun 23;22:523. doi: 10.1186/s12917-026-05651-0 (PMC13555949; doi:10.1186/s12917-026-05651-0)
Supplement: Supplementary file 1 [file 12917_2026_5651_MOESM1_ESM.doc]

**Supplementary materials**

Table S1 The strain information about PCV and FPV

| Virus | GenBank No. | strain | country | time |
| --- | --- | --- | --- | --- |
| CPV | MZ362882.1 | Roseworthy/SA/5371/2019 | Australia | 2019 |
| CPV | KR002798.1 | CPV/CN/JL5/2013 | China | 2013 |
| CPV | MF177233.1 | 19-99 | Italy | 1999 |
| CPV | KM457143.1 | UY364 | Uruguay | 2011 |
| CPV | KR002803.1 | CPV/CN/SD10/2014 | China | 2014 |
| CPV | KY403998.1 | CPV-YH | China | 2008 |
| CPV | MG583676.1 | CPV/CN/ya1/2017 | China | 2015 |
| CPV | KF676668.1 | CPV-JS2 | Chian | 2013 |
| CPV | JQ268283.1 | CPV-LZ1 | Chian | 2011 |
| CPV | OR528754.1 | CPV_UY_252_2a | Uruguay | 2010 |
| CPV | MF177241.1 | Arg9 | Argentina | 2003 |
| CPV | MT629886.1 | CVASU parvovirus1 | Bangladesh | 2018 |
| CPV | MW889113.1 | CPV19 | Peru | 2016 |
| CPV | KT382542.1 | CPV-SH14 | China | 2014 |
| CPV | MF134808.1 | CPV-BJ03/17 | China | 2017 |
| CPV | KF366250.1 | CPV/915-H | India | 2013 |
| CPV | KF638400.1 | s5 | China | 2010 |
| CPV | JX660690.1 | SC02/2011 | China | 2011 |
| CPV | EF011664.1 | B-2004 | China | 2004 |
| CPV | OP957419.1 | CPV-T11 | China | 2016 |
| CPV | PV296089.1 | CPV-2a/Z1-1/200928 | China | 2020 |
| CPV | MW539053.1 | I1 | Turkey | 2020 |
| CPV | OK888554.1 | Arg22 | Argentina | 2013 |
| CPV | MW889101.1 | CPV07 | Peru | 2016 |
| CPV | LC270892.1 | 9985-46 | Japan | 2017 |
| CPV | KR002793.1 | CPV/CN/HB1/2013 | China | 2013 |
| CPV | MZ362881.1 | Emerald/Queensland/4720/2019 | Australia | 2019 |
| CPV | MF177269.1 | E20 | Ecuador | 2011 |
| CPV | OR528750.1 | CPV_PE3_2b | Peru | 2017 |
| CPV | OR528749.1 | CPV_ARG22_2b | Argentina | 2013 |
| CPV | MF177226.1 | 1-99 | Italy | 1999 |
| CPV | ON322759.1 | HN-010 | China | 2020 |
| CPV | MH711894.1 | CU24 | Thailand | 2016 |
| CPV | MW811189.1 | CPV-SH2003 | China | 2020 |
| CPV | KY073269.1 | UFMT | Brazil | 2015 |
| CPV | MF177257.1 | BRA02/14 | Brazil | 2014 |
| CPV | KM457142.1 | UY370c | Uruguay | 2011 |
| CPV | OR528748.1 | CPV_ARG_13_2c | Argentina | 2010 |
| CPV | MF177234.1 | 347-03 | Italy | 2003 |
| CPV | MF510158.1 | CPV_IZSSI_41113_c1_16 | Italy | 2016 |
| CPV | KU508691.1 | HB | Australia | 2015 |
| CPV | MF177266.1 | E13 | Ecuador | 2011 |
| CPV | OM523075.1 | W65 | China | 2021 |
| CPV | MN840830.1 | Canine/SH/1/2019 | China | 2019 |
| CPV | MG013488.1 | CPV-SH1516 | China | 2017 |
| CPV | MK388674.1 | HB2017 | China | 2017 |
| CPV | MT010564.1 | CPV-AHhf1 | China | 2018 |
| CPV | PQ683620.1 | MX-ABM69 | Mexico | 2024 |
| CPV | OR296264.1 | HYD05_2023 | India | 2023 |
| CPV | OP210300.1 | Par2-2022 | Viet Nam | 2022 |
| CPV | MT106232.1 | HCM09 | Viet Nam | 2017 |
| CPV | MH476587.1 | Canine/China/18/2017 | China | 2017 |
| CPV | MT648207.1 | CPV-AHmas9 | China | 2018 |
| CPV | OR451707.1 | YDI1 | Iraq | 2022 |
| CPV | OR528755.1 | CPV2_Vac(NL_35_D) | Uruguay | 2023 |
| CPV | EU659116.1 | CPV-5.us.79 | USA | 1979 |
| CPV | OM640102.1 | LSD39 | Canada | 2018 |
| CPV | MN661243.1 | CPV new 2a variant | India | 2016 |
| CPV | PP035143.2 | CPV-HLJ210620 | China | 2021 |
| CPV | MT165694.1 | NewCPV-2a/China/SC-16/2017 | China | 2017 |
| CPV | MT165692.1 | NewCPV-2b/China/SC-13/2017 | China | 2017 |
| CPV | OR528753.1 | CPV_Uy6_2a | Uruguay | 2006 |
| CPV | OP985292.1 | RCD-PrV/BJF6/red-collared dove/2018 | Chna | 2019 |
| CPV | MN451695.1 | RDPV124 | Finland | 1986 |
| CPV | MN451693.1 | RDPV123 | Finland | 1980 |
| CPV | MN451694.2 | RDPV122 | Finland | 1980 |
| CPV | MN451691.1 | RACCPV1 | USA | 2009 |
| CPV | MN451677.1 | CPV604 | USA | 2007 |
| CPV | MN451674.1 | CPV353 | USA | 1996 |
| CPV | MN451673.1 | CPV307 | USA | 1993 |
| CPV | MN451672.1 | CPV305 | USA | 1993 |
| CPV | MN451671.1 | CPV220 | USA | 1993 |
| CPV | MN451668.1 | CPV63 | USA | 1980 |
| CPV | MN451665.1 | CPV50 | USA | 1986 |
| CPV | MN451654.1 | CPV9 | USA | 1979 |
| CPV | MN451653.1 | CPV6 | USA | 1979 |
| CPV | PV955933.1 | CPV-2c/Wolf/LI9/2022/ITA | Italy | 2022 |
| CPV | PV955932.1 | CPV-new 2a/Wolf/LI8/2021/ITA | Italy | 2021 |
| CPV | PV296090.1 | CPV-2c/Z11/230508 | China | 2023 |
| CPV | KX434460.1 | CPV_IZSSI_52238_12 | Italy | 2012 |
| CPV | KX434456.1 | CPV_IZSSI_45361_09 | Italy | 2009 |
| CPV | KX434454.1 | CPV_IZSSI_29451_09 | Italy | 2009 |
| CPV | JN867617.1 | CPV/Raccoon/349/08 | USA | 2008 |
| CPV | JN867610.1 | CPV/Raccoon/VA/118-A/07 | USA | 2007 |
| CPV | EU659119.1 | CPV-410.us.00 | USA | 2000 |
| CPV | EU659117.1 | CPV-6.us.80 | USA | 1980 |
| CPV | OK888567.1 | UK426 | Uruguay | 2012 |
| CPV | MF177282.1 | UY21 | Uruguay | 2006 |
| CPV | MF177240.1 | 189-02 | Italy | 2002 |
| CPV | MF177239.1 | 288-01 | Italy | 2001 |
| CPV | MF177229.1 | 368-12-17 | Albania | 2012 |
| CPV | MF177225.1 | 242-98 | Italy | 1998 |
| CPV | KM457108.1 | UY82 | Uruguay | 2007 |
| CPV | KM457114.1 | UY169 | Uruguay | 2008 |
| CPV | KM457106.1 | UY55 | Uruguay | 2006 |
| CPV | PQ436980.1 | LG151_dog_Spain | Spain | 2024 |
| FPV | OR602718.1 | ITA/2023/hystrix/213 | Italy | 2023 |
| FPV | PP336908.1 | CVASU/BD/50 | Bangladesh | 2023 |
| FPV | OR921195.1 | FPLV-CC-19-02 | China | 2023 |
| FPV | OR264206.1 | pFPV-sc | China | 2020 |
| FPV | OQ266795.1 | ABT/MVC/2022/FPV/001 | India | 2022 |
| FPV | OM638043.1 | EGY/2021/139-188 | Egypt | 2021 |
| FPV | OM638042.1 | ITA/2021/164-1 | Italy | 2021 |
| FPV | MN908257.1 | MHS2019 | China | 2019 |
| FPV | MH559110.1 | TN/FPV/2018 | India | 2018 |
| FPV | MG924893.1 | GX01 | China | 2016 |
| FPV | KX685354.1 | HN-ZZ1 | China | 2016 |
| FPV | KX900570.1 | HH-1/86 | China | 1986 |
| FPV | KP280068.1 | HRB-CS1 | China | 2014 |
| FPV | PV955934.1 | FPV/Badger/TI6/2022/ITA | Italy | 2022 |
| FPV | PQ436979.1 | LG15_cat_Spain | Spain | 2023 |
| FPV | PV166222.1 | XJ2023 | China | 2023 |
| FPV | PQ227071.1 | XJ-SHZ-2 | China | 2023 |
| FPV | PP419035.1 | FPV/INDIA/MZ30 | India | 2022 |
| FPV | OR528756.1 | FPV_9_AR | Argentina | 2009 |
| FPV | OR727319.1 | FPV-QD22-13 | China | 2022 |
| FPV | OR227624.1 | CH/YCYH/2021 | China | 2021 |
| FPV | OQ863619.1 | YZ2019-027 | China | 2019 |
| FPV | OQ863615.1 | YZ2018-023 | China | 2018 |
| FPV | OM640096.1 | K1830 | Canada | 2018 |
| FPV | MW659466.1 | FPV-SH2002 | China | 2020 |
| FPV | MT178243.1 | SY-01/2019 | China | 2019 |
| FPV | MZ357122.1 | gpfe267par01-12 | China | 2018 |
| FPV | MZ357121.1 | cf093par01-8 | China | 2020 |
| FPV | MW331496.1 | gpfe016par01-5 | China | 2020 |
| FPV | MW811187.1 | FPV-SH2003 | China | 2020 |
| FPV | MN400980.1 | Rara | South Korea | 2017 |
| FPV | MN862749.1 | FPV/River otter/OTVI-3/BC_2019 | Canada | 2019 |
| FPV | MN862746.1 | FPV/American mink/MIVI-21/BC_2017 | Canada | 2017 |
| FPV | MN862745.1 | FPV/American pine marten/MAHG-3/BC_2017 | Canada | 2017 |
| FPV | MN862744.1 | FPV/American pine marten/MAVI-36/BC_2016 | Canada | 2016 |
| FPV | MN862743.1 | FPV/American mink/MIVI-34/BC_2018 | Canada | 2018 |
| FPV | MN127781.1 | 19R124C/TH/2019 | Thailand | 2019 |
| FPV | MN127779.1 | 18R217C/TH/2018 | Thailand | 2018 |
| FPV | MN451692.1 | RACFPV1 | USA | 2011 |
| FPV | MN451652.1 | BFPV | Finland | 1983 |
| FPV | KP019621.2 | TH091305 | Thailand | 2013 |
| FPV | KX434462.1 | FPV_IZSSI_42807_15 | Italy | 2015 |
| FPV | MG764511.1 | FPV-L | China | 2015 |
| FPV | MG764510.1 | FPV-G | China | 1999 |
| FPV | MF069447.1 | FPV/Raccoon/RC18/BC_2016 | Canada | 2016 |
| FPV | MF069446.1 | FPV/Raccoon/RC9/BC_2010 | Canada | 2010 |
| FPV | MF069445.1 | FPV/Raccoon/RC6/BC_2015 | Canada | 2015 |
| FPV | EU659115.1 | FPV-kai.us.06 | USA | 2006 |
| FPV | EU659114.1 | FPV-8b.us_89 | USA | 1989 |
| FPV | EU659112.1 | FPV-4.us_64 | USA | 1964 |
| FPV | EU659111.1 | FVP-3.us_67 | USA | 1967 |
| FPV | EU659113.1 | FPV-8a.us_89 | USA | 1989 |

Table S2 The mean values of RSCU for the four viral genes of CPV

| Synonymous codon (amino acid) | NS1 | NS2 | VP1 | VP2 |
| --- | --- | --- | --- | --- |
| TTT(F) | **1.995** | 1.469 | **1.829** | **1.934** |
| TTC(F) | 0.005 | 0.531 | 0.171 | 0.066 |
| TTA(L) | **2.269** | 1.267 | **2.527** | **2.756** |
| TTG(L) | 1.010 | 0.971 | 1.251 | 1.521 |
| CTT(L) | 1.459 | 1.478 | 1.232 | 0.516 |
| CTC(L) | 0.248 | 0.767 | 0.001 | 0.000 |
| CTA(L) | 0.607 | 1.518 | 0.984 | 1.202 |
| CTG(L) | 0.408 | 0.000 | 0.004 | 0.005 |
| ATT(I) | **1.818** | **1.919** | **2.000** | **2.150** |
| ATC(I) | 0.276 | 1.081 | 0.217 | 0.146 |
| ATA(I) | 0.906 | 0.000 | 0.782 | 0.704 |
| GTT(V) | **1.939** | **1.838** | **1.748** | **1.882** |
| GTC(V) | 0.393 | 0.888 | 0.106 | 0.114 |
| GTA(V) | 0.872 | 0.439 | **1.646** | 1.568 |
| GTG(V) | 0.795 | 0.835 | 0.501 | 0.436 |
| TCT(S) | 1.495 | 1.118 | **2.599** | **2.801** |
| TCC(S) | 0.193 | 1.443 | 0.009 | 0.002 |
| TCA(S) | 0.948 | 1.150 | 1.300 | 1.208 |
| TCG(S) | 0.554 | 0.562 | 0.153 | 0.000 |
| AGT(S) | **2.429** | 0.837 | **1.763** | **1.776** |
| AGC(S) | 0.381 | 0.890 | 0.176 | 0.213 |
| CCT(P) | 1.132 | **1.601** | 1.046 | 1.020 |
| CCC(P) | 0.000 | 0.000 | 0.016 | 0.002 |
| CCA(P) | **2.508** | **2.399** | **2.875** | **2.978** |
| CCG(P) | 0.359 | 0.000 | 0.064 | 0.000 |
| ACT(T) | **1.602** | 1.204 | **1.819** | **1.766** |
| ACC(T) | 0.437 | 1.346 | 0.158 | 0.168 |
| ACA(T) | **1.719** | 1.446 | **1.896** | **1.924** |
| ACG(T) | 0.242 | 0.005 | 0.127 | 0.142 |
| GCT(A) | 0.544 | 0.000 | 1.387 | 1.303 |
| GCC(A) | 0.325 | 1.064 | 0.282 | 0.110 |
| GCA(A) | **2.805** | **2.930** | **2.118** | **2.272** |
| GCG(A) | 0.326 | 0.006 | 0.213 | 0.315 |
| TAT(Y) | 1.551 | 0.851 | **1.694** | **1.772** |
| TAC(Y) | 0.449 | 1.149 | 0.306 | 0.228 |
| CAT(H) | 1.492 | **1.990** | **1.711** | **1.786** |
| CAC(H) | 0.508 | 0.010 | 0.289 | 0.214 |
| CAA(Q) | **1.837** | **1.751** | **1.851** | **1.830** |
| CAG(Q) | 0.163 | 0.249 | 0.149 | 0.170 |
| AAT(N) | 1.363 | 0.853 | 1.444 | 1.507 |
| AAC(N) | 0.637 | 1.147 | 0.556 | 0.493 |
| AAA(K) | 1.458 | 1.200 | **1.782** | **1.891** |
| AAG(K) | 0.542 | 0.800 | 0.218 | 0.109 |
| GAT(D) | 0.967 | 0.839 | **1.653** | **1.862** |
| GAC(D) | 1.033 | 1.161 | 0.347 | 0.138 |
| GAA(E) | **1.780** | 1.179 | **1.630** | 1.587 |
| GAG(E) | 0.220 | 0.821 | 0.370 | 0.413 |
| TGT(C) | **1.847** | 0.495 | **1.949** | **1.953** |
| TGC(C) | 0.153 | 1.505 | 0.051 | 0.047 |
| CGT(R) | 0.013 | 1.289 | 0.189 | 0.259 |
| CGC(R) | 0.738 | 0.430 | 0.362 | 0.000 |
| CGA(R) | 0.246 | 0.430 | 0.032 | 0.044 |
| CGG(R) | 0.249 | 0.000 | 0.187 | 0.259 |
| AGA(R) | **4.736** | **3.852** | **5.038** | **5.433** |
| AGG(R) | 0.018 | 0.000 | 0.191 | 0.005 |
| GGT(G) | **1.669** | 0.000 | **1.651** | **1.763** |
| GGC(G) | 0.512 | 1.324 | 0.185 | 0.222 |
| GGA(G) | **1.689** | **2.666** | 1.483 | 1.493 |
| GGG(G) | 0.130 | 0.009 | 0.681 | 0.522 |

The RSCU values with bold text more than 1.600; the RSCU values with underline less than 0.600.

Table S3 The mean values of RSCU for the four viral genes of FPV

| Synonymous codon (amino acid) | NS1 | NS2 | VP1 | VP2 |
| --- | --- | --- | --- | --- |
| TTT(F) | **1.995** | 1.531 | **1.853** | **1.927** |
| TTC(F) | 0.005 | 0.469 | 0.147 | 0.073 |
| TTA(L) | **2.317** | 1.559 | **2.676** | **2.930** |
| TTG(L) | 0.965 | 0.792 | 1.133 | 1.377 |
| CTT(L) | 1.469 | 1.311 | 1.243 | 0.538 |
| CTC(L) | 0.249 | 0.779 | 0.000 | 0.000 |
| CTA(L) | 0.620 | 1.559 | 0.946 | 1.153 |
| CTG(L) | 0.380 | 0.000 | 0.003 | 0.003 |
| ATT(I) | **1.917** | **2.005** | **2.054** | **2.222** |
| ATC(I) | 0.139 | 0.995 | 0.207 | 0.131 |
| ATA(I) | 0.944 | 0.000 | 0.739 | 0.646 |
| GTT(V) | **1.986** | **1.848** | **1.682** | **1.804** |
| GTC(V) | 0.395 | 0.927 | 0.103 | 0.111 |
| GTA(V) | 0.977 | 0.463 | **1.814** | **1.755** |
| GTG(V) | 0.642 | 0.762 | 0.400 | 0.330 |
| TCT(S) | 1.515 | 1.174 | **2.697** | **2.928** |
| TCC(S) | 0.189 | 1.467 | 0.031 | 0.038 |
| TCA(S) | 0.920 | 1.174 | 1.325 | 1.225 |
| TCG(S) | 0.546 | 0.575 | 0.159 | 0.016 |
| AGT(S) | **2.459** | 0.736 | **1.625** | 1.594 |
| AGC(S) | 0.370 | 0.874 | 0.163 | 0.199 |
| CCT(P) | 1.106 | 1.600 | 1.027 | 0.993 |
| CCC(P) | 0.006 | 0.000 | 0.042 | 0.030 |
| CCA(P) | **2.416** | **2.400** | **2.863** | **2.973** |
| CCG(P) | 0.472 | 0.000 | 0.068 | 0.004 |
| ACT(T) | **1.634** | 1.216 | **1.893** | **1.847** |
| ACC(T) | 0.434 | 1.195 | 0.078 | 0.085 |
| ACA(T) | **1.730** | 1.581 | **1.885** | **1.907** |
| ACG(T) | 0.202 | 0.008 | 0.144 | 0.161 |
| GCT(A) | 0.542 | 0.000 | 1.475 | 1.444 |
| GCC(A) | 0.327 | 1.136 | 0.223 | 0.006 |
| GCA(A) | **2.798** | **2.842** | **2.125** | **2.290** |
| GCG(A) | 0.333 | 0.022 | 0.177 | 0.259 |
| TAT(Y) | 1.529 | 0.838 | **1.785** | **1.925** |
| TAC(Y) | 0.471 | 1.162 | 0.215 | 0.075 |
| CAT(H) | 1.481 | **1.962** | **1.718** | **1.797** |
| CAC(H) | 0.519 | 0.038 | 0.282 | 0.203 |
| CAA(Q) | **1.845** | **1.750** | **1.868** | **1.850** |
| CAG(Q) | 0.155 | 0.250 | 0.132 | 0.150 |
| AAT(N) | 1.357 | 0.868 | 1.516 | 1.588 |
| AAC(N) | 0.643 | 1.132 | 0.484 | 0.412 |
| AAA(K) | 1.472 | 1.272 | **1.826** | **1.909** |
| AAG(K) | 0.528 | 0.728 | 0.174 | 0.091 |
| GAT(D) | 0.987 | 0.800 | **1.628** | **1.811** |
| GAC(D) | 1.013 | 1.200 | 0.372 | 0.189 |
| GAA(E) | **1.766** | 1.173 | **1.700** | **1.667** |
| GAG(E) | 0.234 | 0.827 | 0.300 | 0.333 |
| TGT(C) | **1.849** | 0.506 | **1.935** | **1.942** |
| TGC(C) | 0.151 | 1.494 | 0.065 | 0.058 |
| CGT(R) | 0.000 | 1.405 | 0.191 | 0.265 |
| CGC(R) | 0.743 | 0.402 | 0.382 | 0.011 |
| CGA(R) | 0.250 | 0.419 | 0.000 | 0.000 |
| CGG(R) | 0.237 | 0.000 | 0.199 | 0.276 |
| AGA(R) | **4.770** | **3.774** | **5.032** | **5.448** |
| AGG(R) | 0.000 | 0.000 | 0.196 | 0.000 |
| GGT(G) | **1.640** | 0.000 | 1.574 | **1.672** |
| GGC(G) | 0.496 | 1.338 | 0.192 | 0.231 |
| GGA(G) | **1.692** | **2.636** | 1.505 | 1.509 |
| GGG(G) | 0.172 | 0.026 | 0.729 | 0.589 |

The RSCT valTes with bold text more than 1.600; the RSCT valTes with Tnderline less than 0.600.

Figure S1 Codon neighboring nucleotide context bias of CPV NS1 gene

Figure S2 Codon neighboring nucleotide context bias of CPV NS2 gene

Figure S3 Codon neighboring nucleotide context bias of CPV VP1 gene

Figure S4 Codon neighboring nucleotide context bias of CPV VP2 gene

Figure S5 Codon neighboring nucleotide context bias of FPV NS1 gene

Figure S6 Codon neighboring nucleotide context bias of FPV NS2 gene

Figure S7 Codon neighboring nucleotide context bias of FPV VP1 gene

Figure S8 Codon neighboring nucleotide context bias of FPV VP2 gene

Figure S9 The heatmap for CPV NS1 gene with respect to codon pair usage bias. The grey color means no selection for the corresponding codon pair.

Figure S10 The heatmap for CPV NS2 gene with respect to codon pair usage bias. The grey color means no selection for the corresponding codon pair.

Figure S11 The heatmap for CPV VP1 gene with respect to codon pair usage bias. The grey color means no selection for the corresponding codon pair.

Figure S12 The heatmap for CPV VP2 gene with respect to codon pair usage bias. The grey color means no selection for the corresponding codon pair.

Figure S13 The heatmap for FPV NS1 gene with respect to codon pair usage bias. The grey color means no selection for the corresponding codon pair.

Figure S14 The heatmap for FPV NS2 gene with respect to codon pair usage bias. The grey color means no selection for the corresponding codon pair.

Figure S15 The heatmap for FPV VP1 gene with respect to codon pair usage bias. The grey color means no selection for the corresponding codon pair.

Figure S16 The heatmap for FPV VP2 gene with respect to codon pair usage bias. The grey color means no selection for the corresponding codon pair.
